# Sodium Octanoate Modulates the Innate Immune Response of Bovine Mammary Epithelial Cells through the TLR2/P38/JNK/ERK1/2 Pathway: Implications during *Staphylococcus aureus* Internalization

**DOI:** 10.3389/fcimb.2017.00078

**Published:** 2017-03-15

**Authors:** Nayeli Alva-Murillo, Alejandra Ochoa-Zarzosa, Joel E. López-Meza

**Affiliations:** ^1^Licenciatura en Genómica Alimentaria, Universidad de La Ciénega del Estado de Michoacán de OcampoSahuayo, Mexico; ^2^Facultad de Medicina Veterinaria y Zootecnia, Centro Multidisciplinario de Estudios en Biotecnología, Universidad Michoacana de San Nicolás de HidalgoMorelia, Mexico

**Keywords:** *Staphylococcus aureus*, octanoate, internalization, TLR2, epithelial cells, inflammatory response

## Abstract

Bovine mammary epithelial cells (bMECs) contribute to mammary gland defense against invading pathogens, such as *Staphylococcus aureus* (intracellular facultative), which is recognized by TLR2. In a previous report, we showed that sodium octanoate [NaO, a medium chain fatty acid (C8)] induces (0.25 mM) or inhibits (1 mM) *S. aureus* internalization into bMECs and differentially regulates the innate immune response (IIR). However, the molecular mechanisms have not been described, which was the aim of this study. The results showed that α5β1 integrin membrane abundance (MA) was increased in 0.25 mM NaO-treated cells, but TLR2 or CD36 MA was not modified. When these receptors were blocked individually, 0.25 mM NaO-increased *S. aureus* internalization was notably reduced. Interestingly, in this condition, the IIR of the bMECs was impaired because MAPK (p38, JNK, and ERK1/2) phosphorylation and the activation of transcription factors related to these pathways were decreased. In addition, the 1 mM NaO treatment induced TLR2 MA, but neither the integrin nor CD36 MA was modified. The reduction in *S. aureus* internalization induced by 1 mM NaO was increased further when TLR2 was blocked. In addition, the phosphorylation levels of the MAPKs increased, and 13 transcriptional factors related to the IIR were slightly activated (CBF, CDP, c-Myb, AP-1, Ets-1/Pea-3, FAST-1, GAS/ISRE, AP-2, NFAT-1, OCT-1, RAR/DR-5, RXR/DR-1, and Stat-3). Moreover, the 1 mM NaO treatment up-regulated gene expression of IL-8 and RANTES and secretion of IL-1β. Notably, when 1 mM NaO-treated bMECs were challenged with *S. aureus*, the gene expression of IL-8 and IL-10 increased, while IL-1β secretion was reduced. In conclusion, our results showed that α5β1 integrin, TLR2 and CD36 are involved in 0.25 mM NaO-increased *S. aureus* internalization in bMECs. In addition, 1 mM NaO activates bMECs via the TLR2 signaling pathways (p38, JNK, and ERK1/2), which improves IIR before *S. aureus* invasion. Additionally, NaO (1 mM) might exert anti-inflammatory effects after bacterial internalization.

## Introduction

Medium chain fatty acids (8–14 carbons, MCFAs) are an important energy source present in several foods as medium chain triglycerides (i.e., coconut and palm kernel oil, butter, milk, yogurt, and cheese) (Nagao and Yanagita, 2010). MCFAs possess *in vitro* antimicrobial properties against human gastrointestinal (Marounek et al., 2003; Skrivanova and Marounek, 2007; Aydin et al., 2011) and bovine mastitis pathogens (e.g., *Staphylococcus aureus*) (Nair et al., 2005). In particular, octanoate (8C) has been used in rabbit and poultry farms as a preventive treatment for gastrointestinal diseases (Skrivanova et al., 2008, 2009; Ghareeb et al., 2013). A few studies have described the immunomodulation capacity of octanoate in intestinal epithelial cells (non-professional phagocytic cells, NPPCs), focusing on the inflammatory response (Andoh et al., 2000; Tanaka et al., 2001; Hoshimoto et al., 2002) and the antimicrobial peptide (AP) gene expression (Sunkara et al., 2012; Jiang et al., 2013; Zeng et al., 2013).

The innate immune response (IIR) is a first-line defense mechanism against invading pathogens. Epithelial cells (ECs) participate in this defense through pattern recognition of conserved molecules associated with microorganisms (PAMPs) by means of various families of germ-line-encoded pattern-recognition receptors, including the Toll-like receptors (TLRs) (Bulek et al., 2010). The production of innate immune effectors (enzymes, AP, cytokines, chemokines) is due to the stimulation of ECs through diverse receptors, which in turn leads to the activation of MAPK family proteins (p38, JNK and ERK1/2) and transcription factors (e.g., NF-κB, AP-1, E2F-1, EGR, FAST-1) (Akira et al., 2006; Chiu et al., 2009; Alva-Murillo et al., 2015; Medina-Estrada et al., 2015).

In cattle, bovine mammary epithelial cells (bMECs) are responsible for the production of milk and their response is critical for prompt bacterial clearance and prevention of mastitis (inflammation of the mammary gland) (Brenaut et al., 2014; Thompson-Crispi et al., 2014). *S. aureus* is a major pathogen in many animal species and represents one of the leading causes of clinical and subclinical bovine mastitis, which usually is chronic (Kerro Dego et al., 2002; Scali et al., 2015). This chronicity is related to the ability of *S. aureus* to internalize into professional and NPPCs, such as bMECs (Garzoni and Kelley, 2011; Fraunholz and Sinha, 2012; Scali et al., 2015). In NPPCs, the internalization process depends primarily on the host α5β1 integrin (Hauck et al., 2012; Medina-Estrada et al., 2015). However, other host cell receptors are involved in this process (Alva-Murillo et al., 2014a). In this sense, the blockage of TLR2 in bMECs, the most relevant receptor for *S. aureus* recognition, with neutralizing antibodies decreases the number of internalized bacteria (Alva-Murillo et al., 2015; Medina-Estrada et al., 2015). On the other hand, CD36, a scavenger receptor and a fatty acid translocase, plays a role in *S. aureus* recognition and internalization mainly in professional phagocytic cells by interacting with TLR2 (Hoebe et al., 2005; Stuart et al., 2005). Particularly, the blockage of CD36 with a specific antibody reduces the number of *S. aureus* internalized into bMECs (Alva-Murillo et al., 2015).

The activation of MAPKs that are involved in TLR2 signaling pathways could be regulated by *S. aureus* infection in NPPCs. Thereby, the phosphorylation of p38 is triggered by *S. aureus* (Lamprou et al., 2007; Liang and Ji, 2007; Adhikary et al., 2008; Chekabab et al., 2015; Singh and Kumar, 2015). However, *S. aureus* does not modify the phosphorylation of p38 in osteoblasts and bMECs (Ellington et al., 2001; Alva-Murillo et al., 2015). On the other hand, the bacterial stimulus (live or inactivated) triggers JNK activation (Kumar et al., 2004; Lamprou et al., 2007; Adhikary et al., 2008; Alva-Murillo et al., 2015), while ERK1/2 phosphorylation is reduced by live *S. aureus* in epithelial cells or is stimulated by UV-killed bacteria (Ratner et al., 2001; Adhikary et al., 2008; Alva-Murillo et al., 2015). Additionally, these MAPKs play an important role when *S. aureus* internalizes into bMECs (Alva-Murillo et al., 2015).

The ability of *S. aureus* to internalize into NPPCs allows the bacteria to survive within them, which leads to a low response for conventional antibiotic therapy and induces chronic and recurrent infections (Kerro Dego et al., 2002; Fraunholz and Sinha, 2012). Thus, it is necessary to develop alternative therapies. In this sense, few studies are focused on controlling diseases to prevent pathogen internalization by modulating the host IIR. In a previous study, we demonstrated that sodium octanoate (NaO) exerts a dual effect on *S. aureus* internalization into bMECs because 0.25 mM induces or 1 mM inhibits bacterial invasion (Alva-Murillo et al., 2013). Additionally, NaO (1 mM) favors the AP gene expression during the inhibition of *S. aureus* internalization. However, the molecular mechanisms involved in the NaO modulation of (i) bacterial internalization and (ii) IIRs are unknown. In this study, we showed that α5β1 integrin, TLR2, and CD36 play an important role in 0.25 mM NaO-increased *S. aureus* internalization, but the IIR of bMECs is impaired. On the other hand, 1 mM NaO activates bMECs via TLR2/p38/JNK/ERK1/2 before pathogen invasion, which leads to a better host defense. In addition, this treatment favors an anti-inflammatory response after *S. aureus* infection. Our results highlight the relevance to improve the IIR of mammary glands by using MCFAs, such as NaO, which has a dual effect (concentration dependent) during infection.

## Materials and methods

### *Staphylococcus aureus* strain

*S. aureus* subsp. *aureus* (ATCC 27543) strain was used in this study. This strain was isolated from a case of bovine clinical mastitis and it is able to internalize into bMECs (Gutiérrez-Barroso et al., 2008). Bacteria were grown overnight in Luria-Bertani broth (LB, Bioxon, México). For the different assays the colony forming units (CFU) were adjusted by measuring their optical density at 600 nm (OD 0.2 = 9.2 × 10^7^ CFU/ml).

### Reagents and antibodies

LPS (from *E. coli* 0111:B4) and sodium octanoate (NaO) were acquired from Sigma-Aldrich (St. Louis, MO, USA). In this study, we used 0.25 and 1 mM NaO, which induces or inhibits *S. aureus* internalization into bMECs, respectively (Alva-Murillo et al., 2013). The monoclonal blocking antibodies used were anti-α5β1 integrin (Millipore Cat# MAB2514 Lot# RRID:AB_94626), anti-TLR2 (TL2.1, Abcam Cat# ab9100 Lot# RRID:AB_307008), and anti-CD36 (FA6-152, Abcam Cat# ab17044 Lot# RRID:AB_443600). The MAPK inhibitors SB20358 (p38), SP600125 (JNK), and U0126 (ERK1/2) were acquired from Cell Signaling Technology® (Boston, MA). The working solutions were dissolved in dimethyl sulfoxide (DMSO), which was employed as vehicle in the MAPK activation assay.

### Primary bovine mammary epithelial cells (bMECs) culture

bMECs isolation was performed on udder alveolar tissue from healthy lactating cows as described (Anaya-López et al., 2006). Cells from passages 2–8 were used in all of the experiments. The cells were cultured in petri dishes (Corning-Costar) in growth medium (GM) that was composed of a DMEM medium/nutrient mixture F-12 Ham (DMEM/F-12K, Sigma), which was supplemented with 10% fetal calf serum (Equitech Bio), 10 μg/ml insulin (Sigma), 5 μg/ml hydrocortisone (Sigma), 100 U/ml penicillin, 100 μg/ml streptomycin, and 1 μg/ml amphotericin B (Invitrogen). The bMECs were grown in 5% CO_2_ atmosphere at 37°C.

### Invasion assays

bMECs monolayers were cultivated on 96-well flat-bottom plates (Corning-Costar) that were coated with 6–10 μg/cm^2^ rat-tail type I collagen (Sigma). Prior to the invasion assays, the bMECs (~10 × 10^3^ cells/well) were incubated with 0.25 or 1 mM NaO in DMEM/F12K (Sigma) without antibiotics and serum for 24 h. Then, the cells were treated separately with different blocking antibodies, including anti-α5β1 integrin (10 μg/ml, 30 min), anti-TLR2 (5 μg/ml, 1 h), and anti-CD36 (0.25 μg/ml, 45 min). Mouse or rat IgGs (purified from normal mouse or rat serum that was purchased from Pierce) were used as control. Invasion assays were performed using gentamicin protection assays as described (Gutiérrez-Barroso et al., 2008; Alva-Murillo et al., 2015). Briefly, the bMECs that were used in the antibody blockage experiments were infected with *S. aureus* (MOI 30:1 bacteria per cell) and incubated for 2 h in 5% CO_2_ at 37°C. Then, the cells were washed three times with PBS (pH 7.4) and incubated in GM without serum and penicillin and streptomycin supplemented with 50 μg/ml gentamicin for 1 h at 37°C to eliminate extracellular bacteria. Finally, the bMECs monolayers were detached with trypsin 0.025%–EDTA 0.03% (Sigma) and lysed with 250 μl of sterile distilled water. The cell lysates were plated on LB agar in triplicate and incubated overnight at 37°C. The CFUs were determined with the standard colony counting technique. The data are presented as the ratio CFU/bMECs.

### Membrane abundance (MA) of receptors by flow cytometry analysis

To evaluate the cell-surface expression of the α5β1 integrin, TLR2, and CD36 receptors, ~2 × 10^5^ bMECs/well were cultured at 80% confluence on 24-well plates (Corning-Costar) and were then treated with 0.25 or 1 mM NaO, *S. aureus* or both, as described for the invasion assays. Then, the bMECs were detached with trypsin 0.025%-EDTA 0.03% (Sigma), and the cell pellet was recovered by centrifugation (2,500 rpm, 10 min, 4°C) and was washed 2X with cold-PBS (pH 7.4). The cells were fixed with 4% paraformaldehyde (Sigma) for 10 min at 4°C. The bMECs were blocked with normal goat serum (5% in PBS, Pierce) for 30 min at 4°C with shaking and were then recovered by centrifugation. Furthermore, the bMECs were incubated with the primary antibodies anti-α5β1 integrin (10 μg/ml), anti-TLR2 (0.666 μg/ml in PBS containing 0.1% BSA), or anti-CD36 (2 μg/ml in PBS containing 0.1% BSA) overnight at 4°C. Then, the bMECs were incubated with a FITC-conjugated secondary antibody against rat or mouse IgG (1:50, Molecular Probes) for 2 h on ice. The samples were analyzed in a BD Accuri™ C6 flow cytometer with the BD Accuri C6 Software, and 10,000 events were collected and analyzed. The bMECs that were incubated only with the secondary antibody were used as negative control. For the TLR2 membrane abundance (MA) assays, the positive control consisted in bMECs stimulated with LPS (1 μg/ml, Sigma) for 24 h (Alva-Murillo et al., 2015).

### MAPK activation during NaO-modulated *S. aureus* internalization by flow cytometry

To evaluate the MAPK activation levels by flow cytometry, the bMECs were treated with 0.25 or 1 mM NaO (24 h), *S. aureus* or both, and the samples (30 μg of protein) were prepared according to the manufacturer's protocol for adherent cells (Becton Dickinson, Germany). pp38 (T180/Y182), pJNK1/2 (T183/185), and pERK1/2 (T202/Y204) were quantitatively determined using antibodies from a Flex Set Cytometric Bead Array (Becton Dickinson) according to the manufacturer's protocol. The flow cytometry analyses were performed using the BD Accuri™ C6 and CBA analysis FCAP software (Becton Dickinson). A total of 3,000 events were acquired following the supplied protocol. The minimum detection levels for each phospho-protein were 0.38 U/ml for pJNK and 0.64 U/ml for pp38 and pERK. The negative controls were bMECs treated with pharmacological inhibitors of p38 (5 μM, SB203580), JNK (20 μM, SP600125), or ERK1/2 (2.5 μM, U0126) for 30 min.

### Transcription factor-DNA interactions

Nuclear proteins were obtained from the bMECs using the NE-PER Nuclear and Cytoplasmic Extraction Kit according to the manufacturer's instructions (Thermo Scientific, Rockfor, IL). Protein concentrations were determined with the Bradford assay. The nuclear extracts were subjected to the TranSignal Protein/DNA array I (Panomics, Fremont, CA, USA). Briefly, biotin-labeled DNA-binding oligonucleotides (TranSignal™ Probe Mix) were incubated with 15 μg/ml of nuclear extracts to allow the formation of the transcription factor/DNA complexes. These complexes were separated from the free probes and hybridized to a protein/DNA array; then, the complexes were detected using a HRP-based chemiluminescence method according to the manufacturer's protocol.

### Gene expression analysis by qRT-PCR

To analyze the effects of 0.25 and 1 mM NaO and/or *S. aureus* on the expression of IIR genes in bMECs, monolayers of cells that were cultured in 6-well dishes with 6–10 μg/cm^2^ rat-tail type I collagen (Sigma) were incubated with 0.25 or 1 mM NaO (24 h) and/or *S. aureus* for 2 h (MOI 30:1). bMEC total RNA (5 μg) was extracted with the Trizol reagent (Invitrogen) according to the manufacturer's instructions. Then, the RNA was used to synthesize cDNA as previously described (Alva-Murillo et al., 2013). The expression analysis of the inflammatory response genes was performed with qPCR using the comparative Ct method (ΔΔCt) in a StepOne Plus Real-Time PCR System (Applied Biosystems) according to the manufacturer's instructions. The reactions were carried out with a SYBR Green PCR Master Mix (Applied Biosystems, Carlsbad, CA, USA). Specific primer pairs were acquired from Invitrogen and Elim Biopharm (Table 1), and their specificity was determined by end point PCR. GAPDH was used as an internal control.

**Table 1:** **Bovine oligonucleotides used in this study**.

**Specificity**	**Primer**	**Sequence (5′−3′)**	**Fragment size (bp)**	**Annealing temperature (°C)**	**References**
IL-1β	Forward	GCAGAAGGGAAGGGAAGAATGTAG	198	52	Alva-Murillo et al., 2014b
	Reverse	CAGGCTGGCTTTGAGTGAGTAGAA			
IL-6	Forward	AACCACTCCAGCCACAAACACT	179	57	Alva-Murillo et al., 2014b
	Reverse	GAATGCCCAGGAACTACCACAA			
IL-8	Forward	TTCCACACCTTTCCACCCCAA	149	53.5	Alva-Murillo et al., 2014b
	Reverse	GCACAACCTTCTGCACCCACTT			
IL-10	Forward	GATGCGAGCACCCTGTCTGA	129	59	Alva-Murillo et al., 2014b
	Reverse	GCTGTGCAGTTGGTCCTTCATT			
RANTES	Forward	CACCCACGTCCAGGAGTATT	117	54	Nelson et al., 2010
	Reverse	CTCGCACCCACTTCTTCTCT			
GAPDH	Forward	TCAACGGGAAGCTCACTGG	237	56.9	Yonezawa et al., 2009
	Reverse	CCCCAGCATCGAAGGTAGA			

### Enzyme-linked immunosorbent assay (ELISA)

For the measurement of bovine IL-1β and IL-6 secretions into the medium, the cells (~8 × 10^5^ bMECs/well) were stimulated with NaO (0.25 or 1 mM) and/or challenged with *S. aureus*, as described for the invasion assay, and the conditioned media were collected. The concentrations of both cytokines were measured using the bovine IL-1β and IL-6 screening kits (Thermo Scientific) according to the manufacturer's instructions.

### Analysis of innate immune genes promoter region

In order to evaluate a possible relation between the gene expression levels and the putative binding affinity to transcription factors, the PROMO V 3.0.2 program was used for the analysis of gene promoter region (Messeguer et al., 2002). The putative binding sites were analyzed in a 1.5 kb region upstream of the ATG start site for each gene.

### Data analysis

The data were obtained from three independent experiments each performed in triplicate (except for the ELISA of IL-1β and IL-6, which were performed in duplicated) and compared with Student's *t*-test and analysis of variance (ANOVA). The results are reported as the means ± the standard errors (SE), and the significance level was set at *P* < 0.05, except for RT-qPCR analysis where fold-change values greater than 2 or less than 0.5 were considered as significant differentially expressed mRNAs (Morey et al., 2006). For gene expression and membrane staining assays data were normalized to the untreated cells (control).

## Results

### α5β1 integrin, TLR2, and CD36 are involved in NaO-induced *S. aureus* internalization into bMECs, but only TLR2 is implied in NaO-reduced bacterial internalization

**Figure 1:**
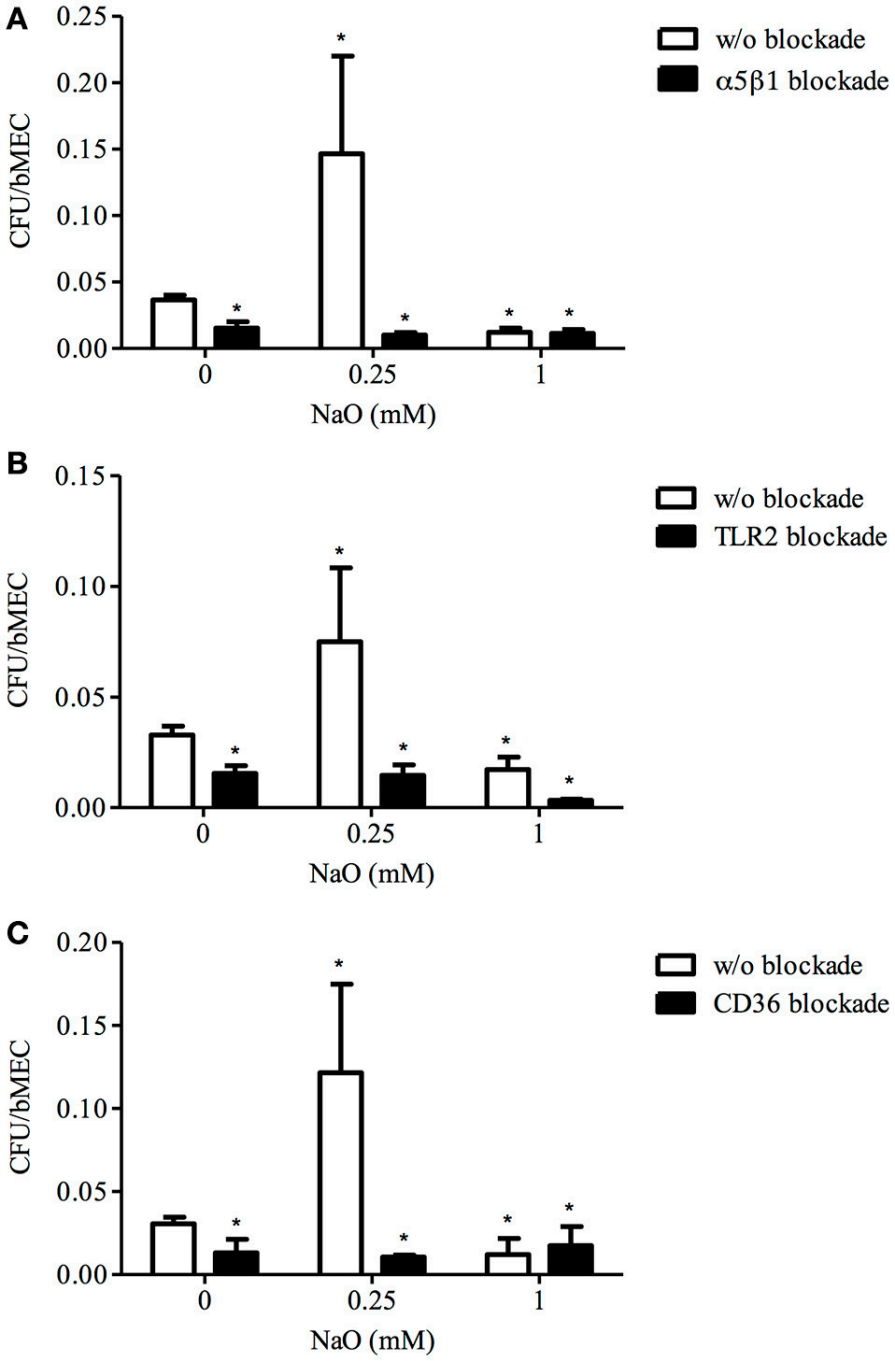
**The role of α5β1 integrin, TLR2, and CD36 in *S. aureus* internalization into bMECs modulated by NaO**. bMECs were treated with 0.25 or 1 mM NaO for 24 h, then incubated with a specific blocking **(A)** anti-α5β1 integrin (10 μg/ml), **(B)** anti-TLR2 (5 μg/ml), or **(C)** anti-CD36 (0.25 μg/ml) antibody for 30 min, 1 h, or 45 min, respectively, and further challenged with *S. aureus* for 2 h. The number of internalized bacteria is represented by the ratio CFU/bMEC. Each bar shows the mean of triplicates ± SE of three independent experiments. The symbol “^*^”indicates significant changes (*P* < *0.05*) compared to control cells (untreated cells and w/o blockade).

Previously, we demonstrated that the *S. aureus* internalization is increased in the 0.25 mM NaO-treated bMECs; whereas, for the 1 mM NaO treatment, the number of *S. aureus* recovered is reduced (Alva-Murillo et al., 2013). In addition, it is known that α5β1 integrin, TLR2, and CD36 play an important role in the *S. aureus* internalization into bMECs (Alva-Murillo et al., 2015; Medina-Estrada et al., 2015). However, the participation of these receptors in the NaO mediated-internalization process is unknown. To determine the roles of these receptors, bMECs were treated with 0.25 or 1 mM NaO (24 h), and the receptors were blocked independently prior to the *S. aureus* challenge. As expected, 0.25 and 1 mM NaO induced (~2-fold) and reduced (~50%) the bacterial internalization, respectively. As we reported previously, the bacterial internalization decreased (~50%) when α5β1 integrin, TLR2, or CD36 were blocked with specific antibodies (Figure 1). Interestingly, the blockage of α5β1 integrin, TLR2, or CD36 after 0.25 mM NaO treatment considerably reduced the NaO-induced *S. aureus* internalization (0.01, 0.014, and 0.01 ratios, respectively) (Figure 1). On the other hand, the *S. aureus* internalization was dramatically decreased (0.003 ratio) in the 1 mM NaO-treated bMECs after TLR2 blockade because the CFU recovered were even lesser than in the 1 mM NaO-treated cells (0.017 ratio) (Figure 1B), the difference between both treatments was statistically significant (w/o blockade and TLR2 blockade, *P* < *0.05*). Additionally, in the same condition, the blockage of α5β1 integrin or CD36 did not modify the effect of this MCFA (Figures 1A,C). Of note, these effects were specific because the bacterial internalization was not modified in mouse or rat IgG-treated cells (data not shown). These results suggest that α5β1 integrin, TLR2, and CD36 are involved in the 0.25 mM NaO-increased *S. aureus* internalization into bMECs. Meanwhile, only TLR2 seems to play an important role in 1 mM NaO-reduced bacterial internalization.

### The α5β1 integrin membrane abundance (MA) is induced by 0.25 mM NaO and TLR2 MA is increased by 1 mM NaO

**Figure 2:**
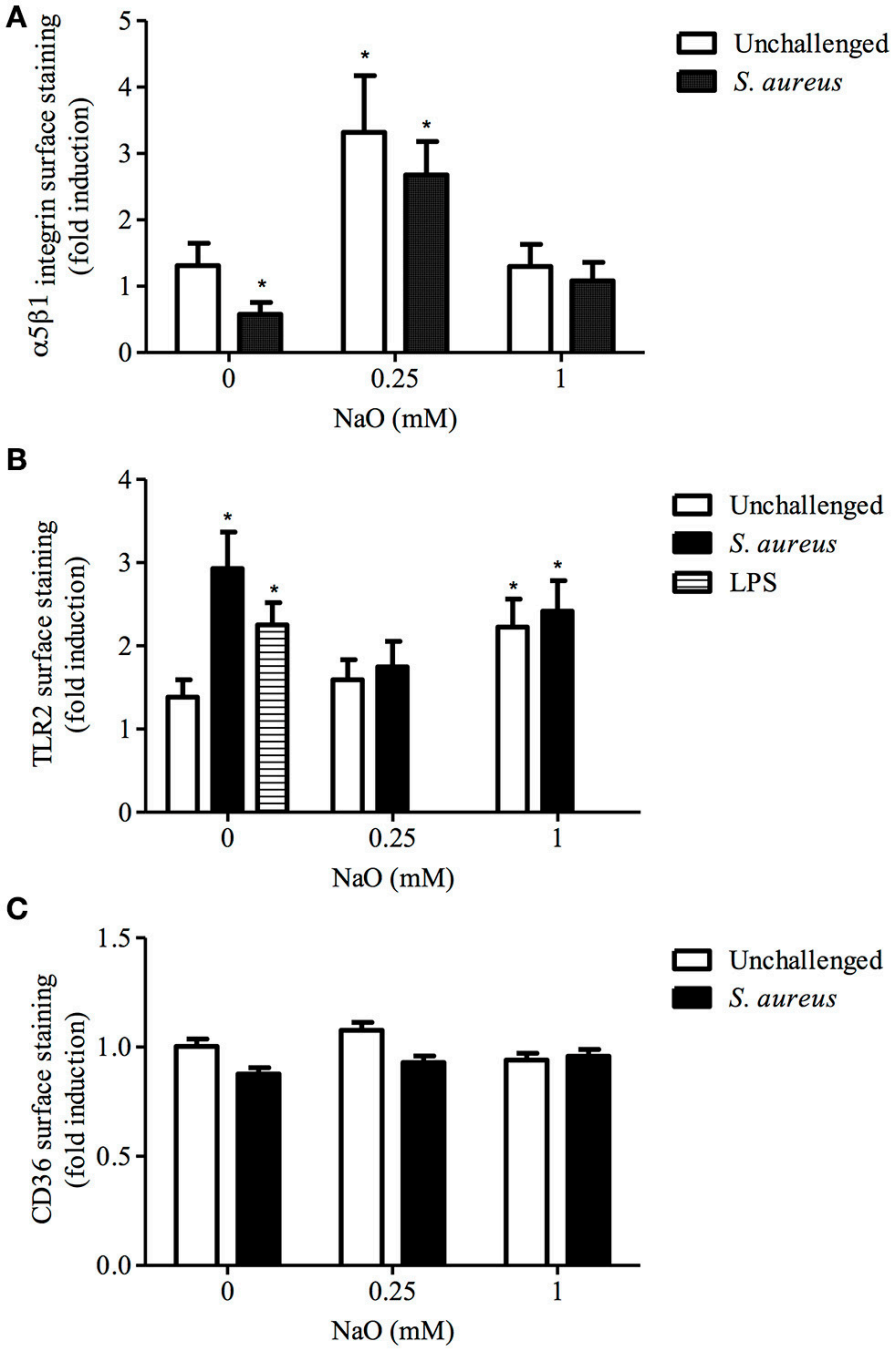
**The α5β1 integrin, TLR2, and CD36 membrane abundance (MA) regulated by NaO**. bMECs were treated with 0.25 or 1 mM NaO (24 h) and/or challenged with *S. aureus* and the **(A)** α5β1 integrin, **(B)** TLR2, and **(C)** CD36 receptor abundance was evaluated by flow cytometry. The fluorescence intensity was estimated from 10,000 events. For TLR2 MA, LPS was used as a positive control. Each bar shows the mean of triplicates ± SE of three independent experiments. The symbol “^*^” indicates significant changes (*P* < 0.05) compared to control cells (untreated cells).

Next, we explored whether there is a correlation between the blocking assays and receptor membrane abundance (MA). As we reported previously, *S. aureus* decreases the α5β1 integrin MA and induces the TLR2 MA in bMECs, but does not alter the CD36 MA (Alva-Murillo et al., 2015; Medina-Estrada et al., 2015). The α5β1 integrin MA was increased (~2.5-fold) by 0.25 mM NaO treatment, and this induction did not change after bacterial stimulus (Figure 2A). In addition, the TLR2 and CD36 MA were not modified (Figures 2B,C), and these data correlated with the gene expression of both receptors (data not shown). Interestingly, 1 mM NaO (24 h) augmented the TLR2 MA (~2-fold), and this level was maintained after *S. aureus* challenge (Figure 2B). A similar response was observed in the TLR2 mRNA levels; however, it was not statistically significant (data not shown). In this condition, neither the CD36 MA (Figure 2C) nor the expression of its gene were modified (data not shown). According to these results, we propose that 0.25 mM NaO might induce the *S. aureus* internalization into bMECs through α5β1 integrin; meanwhile, 1 mM NaO can activate bMECs via TLR2 before *S. aureus* challenge.

### MAPK activation by NaO

**Figure 3:**
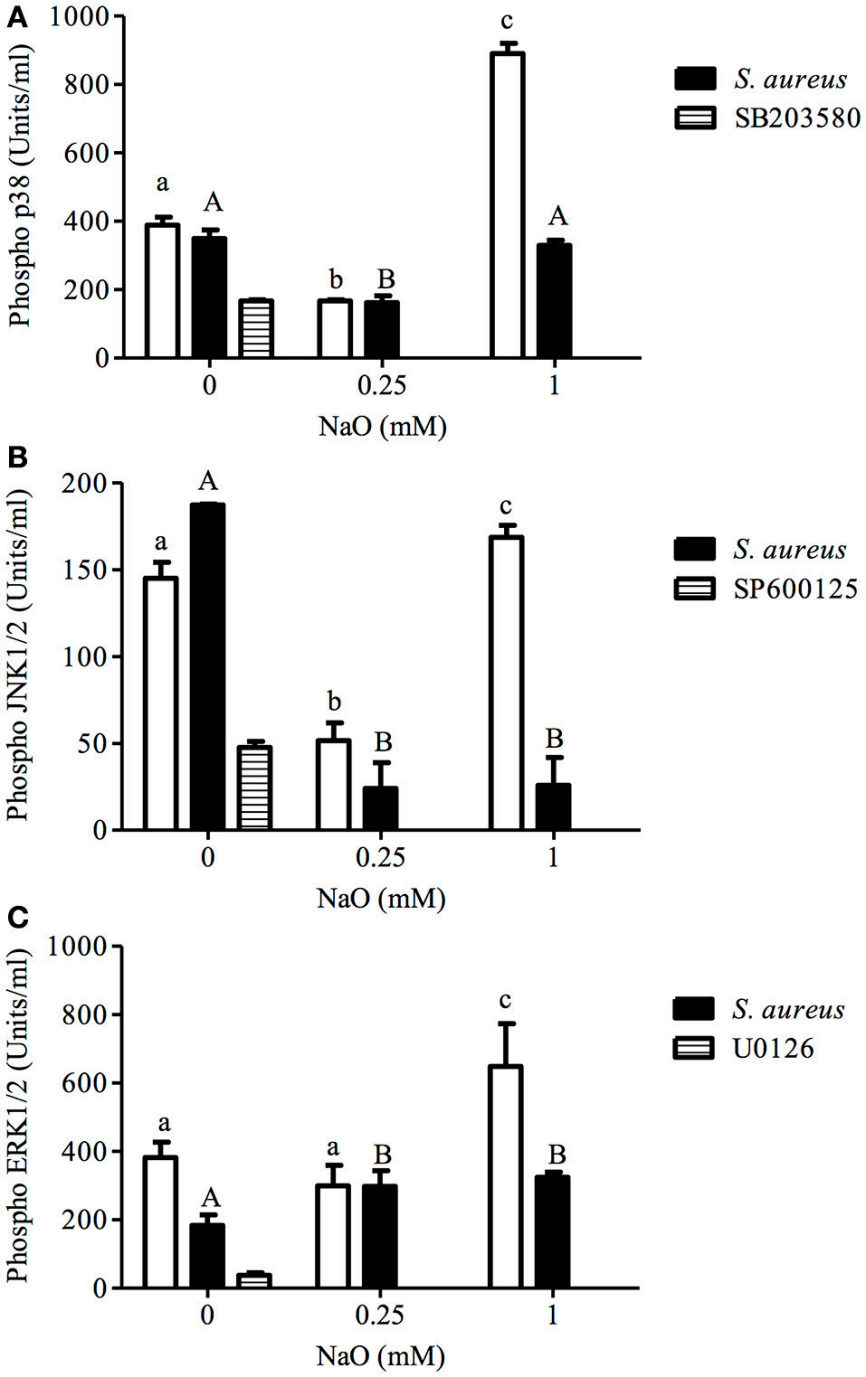
**p38, JNK, and ERK1/2 activation regulated by NaO in *S. aureus*-challenged bMECs**. MAPK phosphorylation was measured in bMECs that were treated with 0.25 or 1 mM NaO and/or challenged with *S. aureus* by flow cytometry. The phosphorylated MAPK concentrations (U/ml) are represented: **(A)** pp38, **(B)** pJNK1/2, and **(C)** pERK1/2. Each bar shows the result of one experiment. SB203580: p38 inhibitor. SP600125: JNK1/2 inhibitor. U0126: ERK1/2 inhibitor. Different letters above the bars indicate significant changes among the unchallenged bMECs (white bars) and *S. aureus*-challenged cells within the same MAPK evaluated (*p* < 0.05).

Because TLR2 activation leads to MAPK phosphorylation (p38, JNK, or ERK1/2), and these kinases are involved in bacteria phagocytosis in both phagocytic and NPPCs (Ninkovic and Roy, 2012; Alva-Murillo et al., 2015), we evaluated the phosphorylation status of these MAPKs in NaO-treated cells. As expected, the JNK phosphorylation level was slightly increased (1.3 ratio) in the *S. aureus*-challenged bMECs, but the activation of ERK1/2 was reduced (0.48 ratio), and the p38 activation was not modified (Figure 3). When the bMECs were treated with 0.25 mM NaO (24 h), the basal activation of p38, JNK, and ERK1/2 decreased (0.57, 0.65, and 0.22-fold, respectively), and this effect was not modified after *S. aureus* challenge, except for JNK where the inhibition was strengthened (0.84-fold). The p38 and JNK phosphorylation reduction in the 0.25 mM NaO-treated bMECs was similar to that observed in cells treated with pharmacological inhibitors (Figures 3A,B). Interestingly, 1 mM NaO increased the phosphorylation of the three MAPKs being more evident for p38 (~2-fold). However, after infection, the stimulatory effect of 1 mM NaO was reverted to basal levels, except for JNK1/2, where the phosphorylation was even lower. Taken together, these results suggest that 0.25 mM NaO inhibits the bMECs IIR because the MAPK phosphorylation status was reduced, whereas 1 mM NaO favors the activation of the bMECs through p38, JNK, and ERK1/2 phosphorylation.

### Activation of transcription factors (TFs) related to the innate immune response (IIR)

**Figure 4:**
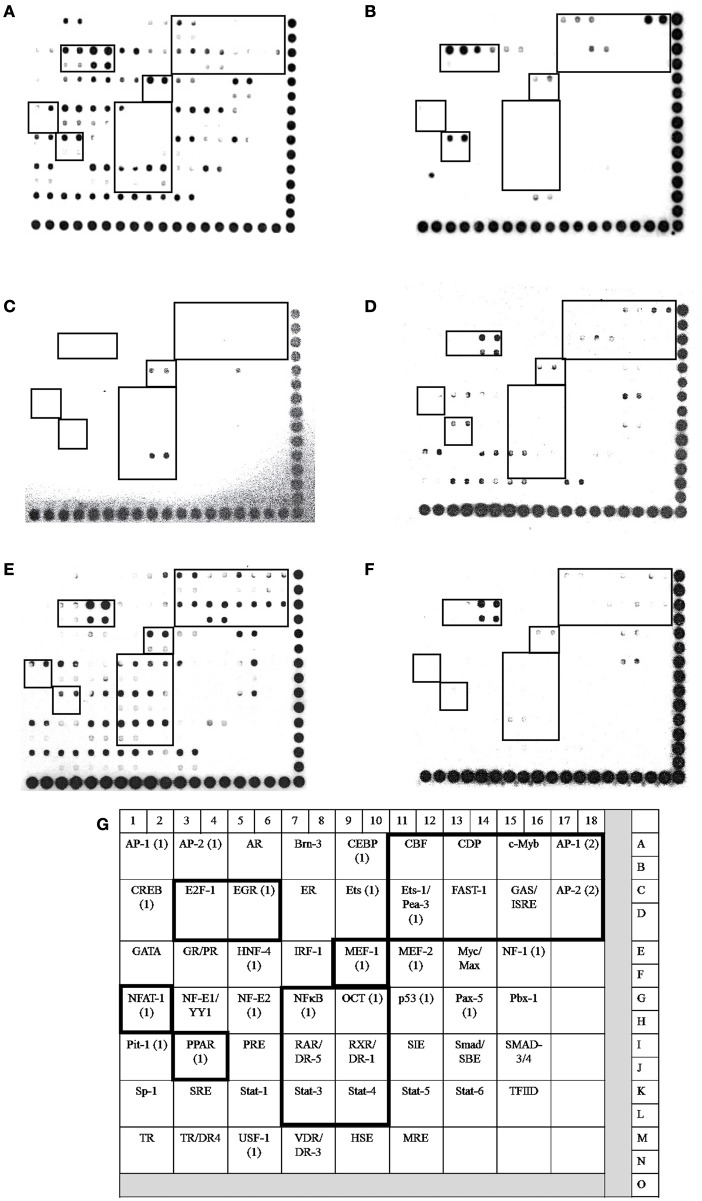
**Transcription factor activation by NaO in *S. aureus*-challenged bMECs**. Protein/DNA array blots were used to analyze 56 different transcription factor DNA-binding sites from samples that were obtained from **(A)** bMEC nuclear extracts (control), **(B)** bMECs that were challenged with *S. aureus*, **(C,E)** bMECs that were treated with 0.25 or 1 mM NaO for 24 h, and **(D,F)** cells that were treated with 0.25 or 1 mM NaO and challenged with *S. aureus* for 2 h. **(G)** Schematic diagram of transignal™ protein/DNA array I. The DNA samples were spotted in duplicate in two rows (top: Undiluted; bottom: Dilution 1/10). Biotinylated DNA was spotted for alignment along the right and bottom sides of the array. TFs whose activation status was affected by NaO are highlighted with boxes. AP-1, activating-protein 1; AP-2, activating enhancer binding protein 2; CBF, core binding factor; CDP, CCAAT displacement protein; c-Myb, c-myeloblastosis transcription factor; DR, death receptor; EGR-1, early growth response protein 1; Ets-1, Ets-1 transcriptional factor; E2F-1, E2F transcription factor 1; FAST-1, forkhead activin signal transducer-1; GAS, interferon-gamma activated sequence; ISRE, interferon-stimulated response element; MEF-1, myeloid Elf-1 like factor; NF-κB, nuclear factor-κB; NFAT-1, nuclear factor or activated T cells; PPAR, peroxisome proliferator-activated receptor; OCT-1, octamer transcription factor; RAR, retinoic acid receptor; RXR, retinoid X receptor; Stat, signal transducer and activator of transcription.

**Figure 5:**
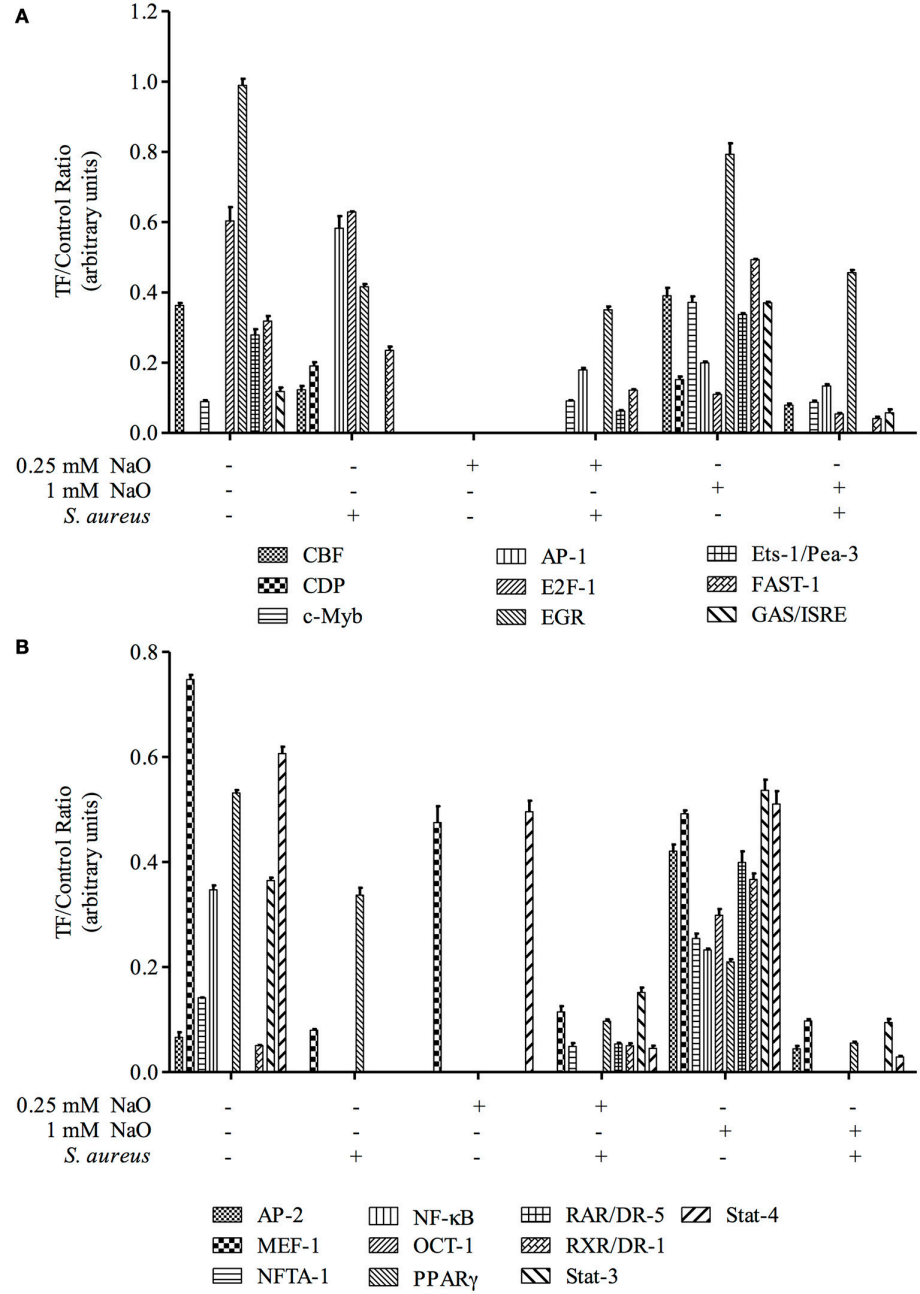
**Transcription factor activation analysis**. The intensity of each spot from the membranes was quantified using ImageJ software (ImageJ, RRID:SCR_003070). The bars show the mean intensity from duplicates of the ratio TF/control.

In bMECs, TLR2 activation triggers signaling pathways that induce the activation of transcriptional factors (TFs) related to host defense, such as AP-1, NF–κB, E2F-1, FAST-1, MEF-1, EGR, PPAR, ER, and CBF, among others (Alva-Murillo et al., 2015; Bauer et al., 2015; Medina-Estrada et al., 2015). In addition, NaO modulates TF activation related to the immune response (PPARγ, C/EBPα) (Han et al., 2002; Yonezawa et al., 2004; Yang et al., 2009). In this sense, we evaluated the activation status of 56 TFs related to IIR by a Protein/DNA array (Figures 4, 5). The bMECs exhibited a basal activation profile where 42 TF were activated (ratio of 0.05–0.98) showing the TF EGR, MEF-1, Stat-1, NF-E1/YY, Stat-4, and E2-F1 the strongest activation (0.98, 0.75, 0.7, 0.65, 0.61, 0.6 ratio, respectively). Interestingly, the basal activation was reduced after *S. aureus* challenge, but only AP-1 and E2F1 displayed an evident signal (0.58 and 0.63 ratio, respectively) (Figures 4B, 5A). Intriguingly, in the 0.25 mM NaO-treated bMECs, the basal activation was drastically inhibited after *S. aureus*-challenge (Figures 4C,D, 5). In this condition, the TF MEF-1 and Stat-4 were activated (0.47 and 0.5 ratio, respectively), nevertheless, this response was lesser than the basal status. Additionally, 1 mM NaO treatment slightly activated (from a ratio of 0.15 to 0.53) 13 TFs (CBF, CDP, c-Myb, AP-1, Ets-1/Pea-3, FAST-1, GAS/ISRE, AP-2, NFAT-1, OCT-1, RAR/DR-5, RXR/DR-1, and Stat-3) in relation to unchallenged cells. The basal state of EGR was reduced (0.79 ratio) in the 1 mM NaO-treated cells, and nevertheless, it was the highest TF activation (Figure 4E). This effect was abolished by infection since the activation status of 45 TF (Brn-3, CEBP, CBF, CDP, c-Myb, AP-1, CREB, E2F-1, EGR, ER, Ets, Ets-1/Pea-3, FAST-1, GAS/ISRE, AP-2, GR/PR, HNF-4, IRF-1, MEF-1, MEF-2, NF-1, NFTA-1, NF-E1/YY, NF-E2, NFκB, OCT, p53, PPAR, PRE, RAR/DR-5, RXR/DR-1, SMAB-3/4, Sp-1, SRE, Stat-1, Stat-3, Stat-4, Stat-5, Stat-6, TR, TR/DR-4, USF-1, VDR/DR-3, HSE, and MRE) were decreased in 1 mM NaO-treated bMECs after *S. aureus* challenge in relation to 1 mM NaO treated cells (Figures 4E,F, 5). Markedly, the basal status of NF-κB and PPARγ was reduced in all of the conditions evaluated. These results suggest that (i) 0.25 mM NaO impairs the IIR of bMECs, and (ii) 1 mM NaO activates bMECs via TLR2 prior to infection.

### Gene expression and secretion of inflammatory response elements

**Figure 6:**
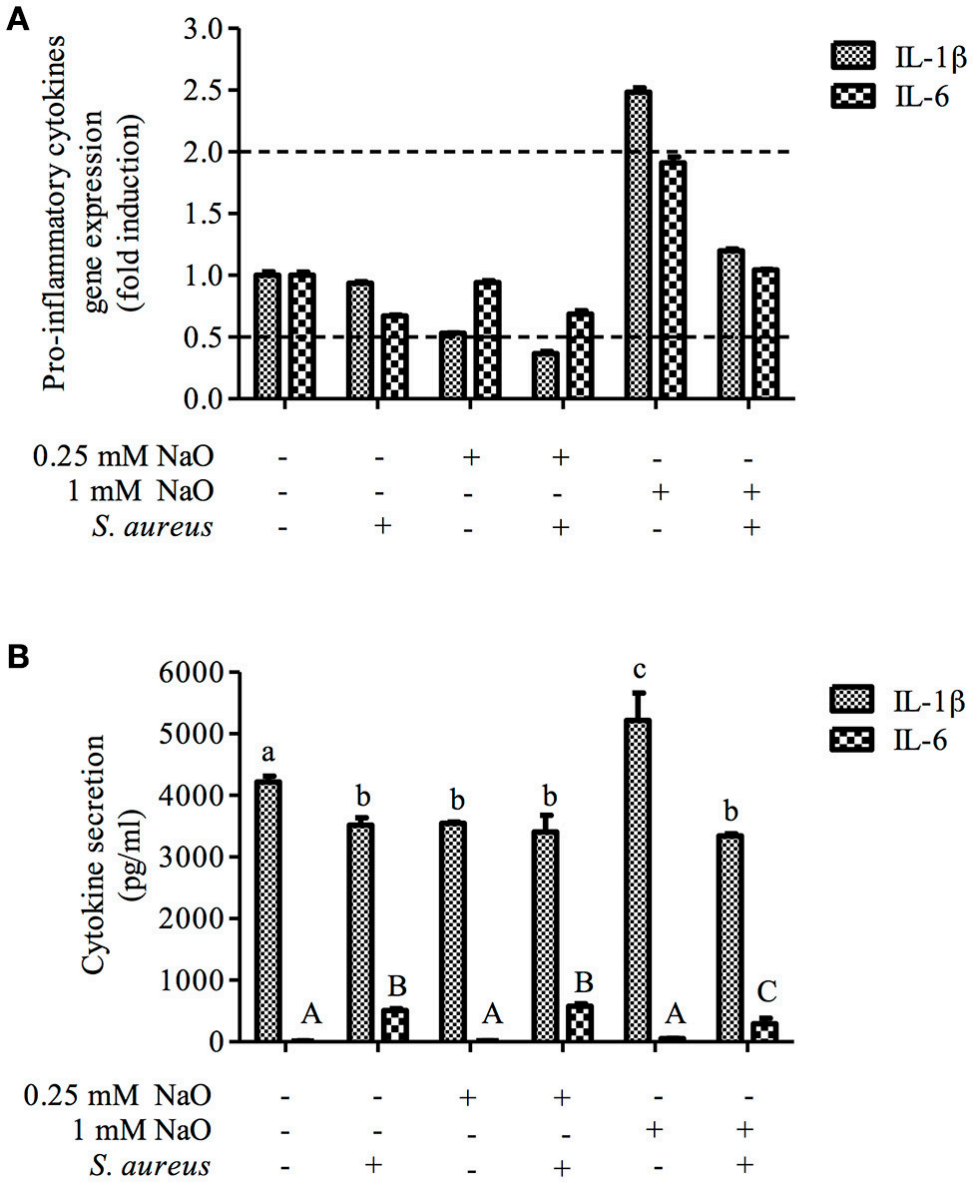
**The gene expression and protein secretion of pro-inflammatory cytokines modulated by NaO. (A)** qPCR analysis showing the mRNA levels of IL-1β and IL-6. The bMECs were treated with 0.25 or 1 mM NaO and then challenged with *S. aureus* for 2 h. Each bar shows the mean of triplicates ± SE of three independent experiments. GAPDH was used as endogenous gene in all of the conditions. Fold-change values greater than 2 or less than 0.5 were considered as significant differentially expressed mRNAs. **(B)** The concentration of IL-1β and IL-6 in culture medium of bMECs treated with NaO (0.25 or 1 mM) for 24 h and then challenged with *S. aureus* for 2 h. The protein concentrations were determined by ELISA. Different letters above the bars indicate significant changes among the treatments within the same cytokine evaluated (*P* < 0.05). IL-1β, interleukin-1beta; IL-6, interleukin-6.

**Figure 7:**
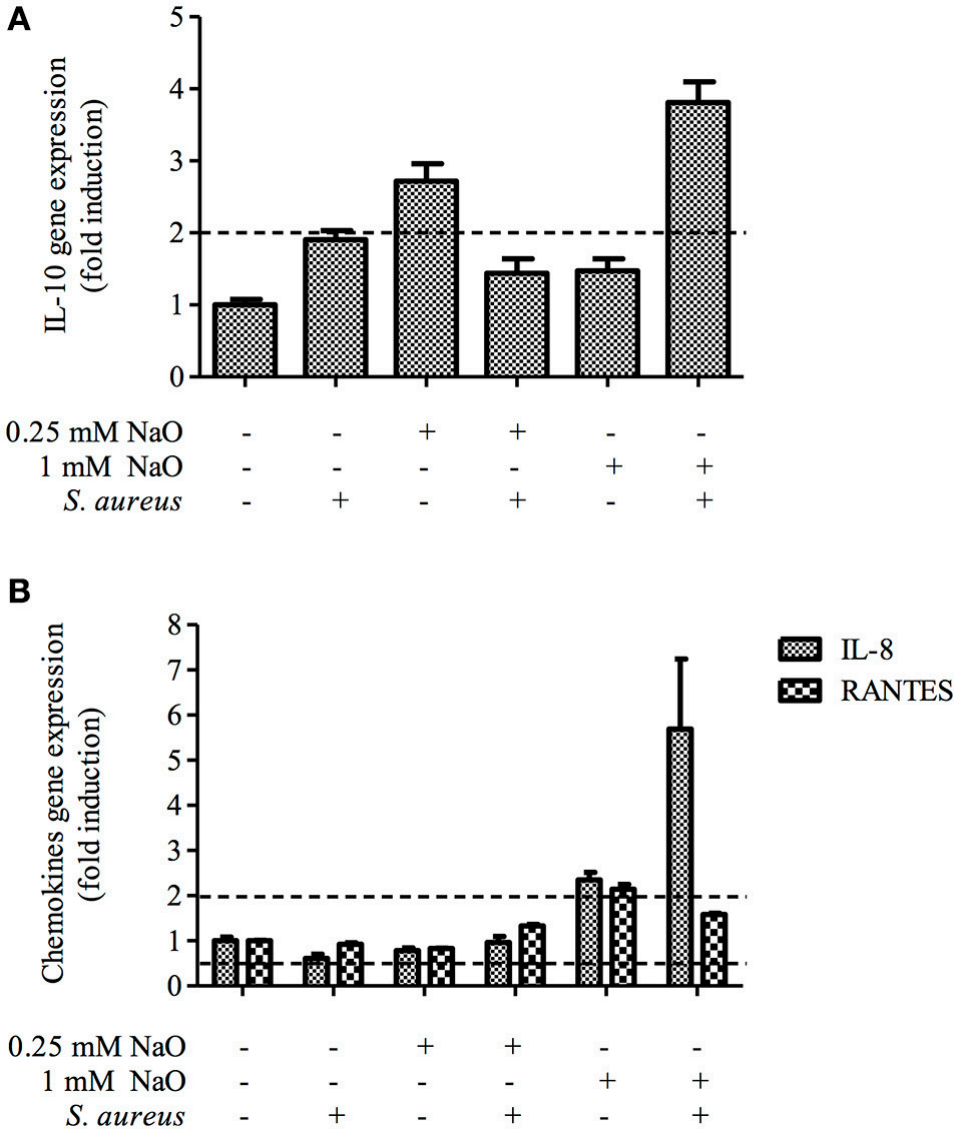
**Expression of anti-inflammatory cytokines and chemokines in bMECs treated with NaO and infected with *S. aureus***. qPCR analysis that shows the effect on anti-inflammatory cytokine **(A)** and chemokines **(B)** mRNA levels. The bMECs were treated with 0.25 or 1 mM NaO and then challenged with *S. aureus* for 2 h. Each bar shows the mean of triplicates ± SE of three independent experiments. GAPDH was used as endogenous gene in all of the conditions. Fold-change values greater than 2 or less than 0.5 were considered as significant differentially expressed mRNAs. IL-10, interleukin-10; IL-8, interleukin-8; RANTES, Regulated on Activation, Normal T Expressed and Secreted.

The NaO-modulated antimicrobial response was evaluated previously by measuring the gene expression of antimicrobial peptides (TAP, LAP, BNBD4, BNBD5, and BNBD10), as well as the mRNA levels of the proinflammatory cytokine TNF-α (Alva-Murillo et al., 2013). However, a broader study to determine the effect of NaO on the inflammatory response of bMECs has not been performed. To achieve this, we evaluated the gene expression of pro-inflammatory (IL-1β and IL-6) and one anti-inflammatory cytokine (IL-10), as well as 2 chemokines (IL-8 and RANTES), in NaO-treated bMECs during infection. Regarding the pro-inflammatory cytokines, we performed an ELISA to the evaluate protein secretion. *S. aureus* did not change the mRNA level of any gene evaluated (Figures 6, 7). The 0.25 mM NaO treatment increased the IL-10 gene expression (~3-fold), but this effect was reduced after infection (Figure 7A). The 1 mM NaO-treated bMECs showed an induction of IL-1β (~2.5-fold), IL-8 (~2.4-fold), and RANTES (~2-fold) mRNA levels, which were reverted by *S. aureus*, except for IL-8, where the level increased to ~6-fold (Figures 6, 7). In addition, the IL-10 mRNA level was not modified in the cells treated with 1 mM NaO. However, after infection the mRNA level was up-regulated (~4-fold) (Figure 7A).

With regard to protein secretion, the concentration of IL-1β in the supernatant was slightly reduced in the *S. aureus*-challenged bMECs in relation to the unstimulated cells, but the IL-6 secretion was increased (Figure 6B). The 0.25 mM NaO treatment diminished the secretion of IL-1β, and this effect was maintained after *S. aureus* challenge (Figure 6B). A similar response was observed at the mRNA level (Figure 6A). When the 0.25 mM NaO-treated cells where infected with *S. aureus*, the IL-6 secretion was increased dramatically (~42-fold) in relation to the control cells. On the other hand, the 1 mM NaO treatment augmented the IL-1β secretion (5,218 pg/ml), but this effect was reverted by *S. aureus* challenge (3,343 pg/ml), and this reduction was even lower than in the control cells (4,218 pg/ml). Interestingly, these results correlate with the mRNA levels. Additionally, the IL-6 secretion was not modified in the 1 mM NaO-treated cells. However, after infection this response was augmented (~21-fold) in relation to the unchallenged cells (Figure 6B). Considering these results, we suggest that 0.25 mM NaO acts as an anti-inflammatory due to the up-regulation of IL-10 gene expression and IL-1β secretion. Meanwhile, 1 mM NaO may exert pro-inflammatory effects prior to infection, with respect to IL-1β gene expression and secretion as well as chemokine gene expression, but it can act as an anti-inflammatory after infection due to the IL-10 and IL-1β gene expression and the IL-1β secretion.

### Analysis of the promoter regions of the antimicrobial peptides and the cytokine genes

**Figure 8:**
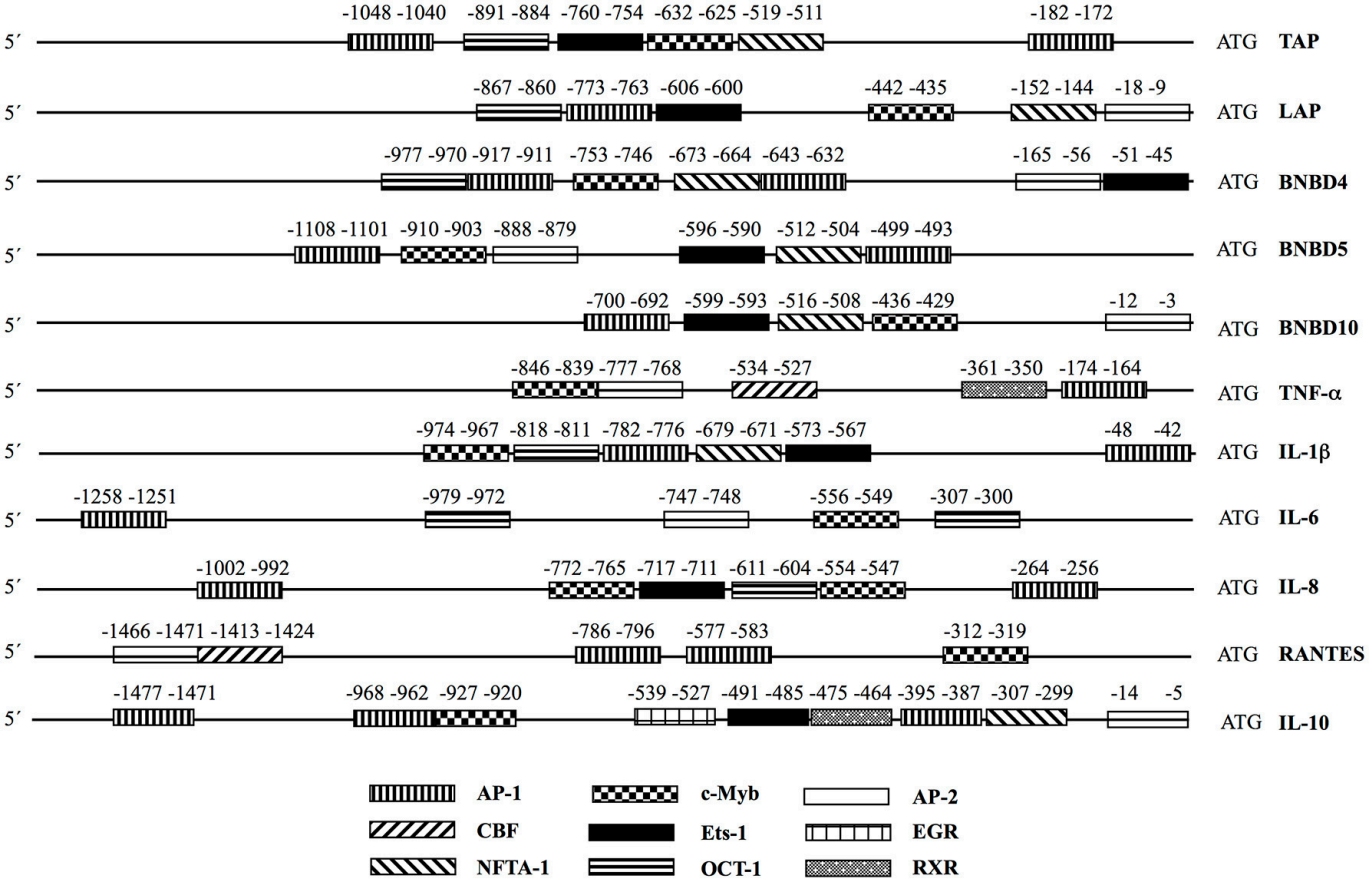
**Transcription binding site analysis in the promoters of innate immune elements**. The program PROMO V 3.0.2 was used. The putative binding sites were analyzed in 1.5 kb upstream of the ATG in each gene. TAP, tracheal antimicrobial peptide (Genbank_AC_000184.1); LAP, lingual antimicrobial peptide (Genbank_AC_000184.1); BNBD4, bovine neutrophil β-defensin 4 (Genbank_AC_000184.1); BNBD5, bovine neutrophil β-defensin 5 (Genbank_AC_000184.1); BNBD10, bovine neutrophil β-defensin 10 (Genbank_AC_000184.1); TNF-α, tumor necrosis factor-alpha (Genbank AC_000180.1); IL-1β, interleukin-1beta (Genbank AC_000168.1); IL-6, interleukin-6 (Genbank AC_000161.1); IL-10, interleukin-10 (Genbank AC_000173.1); RANTES, Regulated on Activation, Normal T Expressed and Secreted; IL-8, interleukin-8 (Genbank AC_000163.1).

To determine whether the last results might be related with the activation of TFs, we carried out an analysis of the promoter regions of the antimicrobial peptides (Alva-Murillo et al., 2013) and the cytokine genes evaluated to find putative transcription binding sites for the factors that were activated by NaO (Figure 8). Interestingly, all of the evaluated genes showed putative transcription binding sites for AP-1 and c-Myb, which might explain their induction in gene expression by 1 mM NaO in bMECs through the TLR2 pathway. Although EGR showed the strongest activation in this condition, only the IL-10 gene showed a putative binding site for this TF. We did not find putative transcriptional binding sites for the others TFs activated by 1 mM NaO (CDP, FAST-1, GAS-ISRE, RAR/DR-5, Stat-3).

## Discussion

*S. aureus* can internalize into phagocytic cells and NPPCs (i.e., bMECs), which avoid the host immune response and favors chronic and recurrent infections (i.e., bovine mastitis). The modulation of the IIR is essential for the improvement of prophylactic or therapeutic treatments to avoid diseases in animals or humans. In this way, NaO regulates the inflammatory and antimicrobial response in intestinal epithelial cells (Andoh et al., 2000; Tanaka et al., 2001; Hoshimoto et al., 2002; Sunkara et al., 2012; Jiang et al., 2013; Zeng et al., 2013). However, little is known about the role of this fatty acid during infection diseases caused by invading pathogens. Previously we showed that NaO differentially modulates the *S. aureus* internalization into bMECs and the antimicrobial response (Alva-Murillo et al., 2013). However, the molecular mechanisms of the *S. aureus* internalization into bMECs and the inflammatory response modulated by NaO are unknown. This study demonstrates that (i) in 0.25 mM NaO-treated bMECs, the IIR is not mediated by TLR2, and its signaling pathway (MAPKs and TF) is impaired, and (ii) 1 mM NaO treatment activates bMECs via the TLR2/p38/JNK/ERK1/2 pathway prior to infection, which might be involved in the 1 mM NaO-reduced *S. aureus* internalization into bMECs.

The main mechanism described for *S. aureus* internalization into bMECs is mediated by the α5β1 integrin (Alva-Murillo et al., 2014a). Interestingly, the blockage of this receptor drastically reduced the 0.25 mM NaO-increased *S. aureus* internalization but did not alter the 1 mM NaO-reduced bacterial internalization. In addition, we observed an effect dependent of NaO concentration on the integrin MA in bMECs, 0.25 mM NaO (24 h) increased MA (~3-fold) but 1 mM NaO did not modify it (Figure 2A). In same way, *S. aureus* internalization into bMECs induced by prolactin correlated with an increase of α5β1 integrin MA (Medina-Estrada et al., 2015). In contrast, the membrane adundance of β1 integrin was not affected by NaO (1 mM, 24 h) in human bladder cancer cells (Yamasaki et al., 2014). These results suggest that the α5β1 integrin availability might favor the 0.25 mM NaO-induced *S. aureus* internalization. However, this receptor is not directly involved in the 1 mM NaO-reduced bacterial internalization.

TLRs are part of the first-line defense mechanisms of epithelial cells. In particular, TLR2 participates in *S. aureus* recognition. When TLR2 is blocked with a functional antibody (clone TL2.1), the number of CFU recovered into human cord blood-derived cells or bMECs is reduced (Rocha-de-Souza et al., 2008; Medina-Estrada et al., 2015). We observed similar results in our conditions (Figure 1B). There are few reports regarding the effect of medium chain fatty acids on TLR expression. In this sense, rats fed with a high-fat diet show increased levels of TLR2 gene and protein expression in skeletal muscle (Zhu et al., 2015). However, a diet based on medium-chain triglycerides does not modify the level of TLR2 mRNA in a rat colitis model (Mañé et al., 2009). Interestingly, we only detected an increase in the TLR2 MA in 1 mM NaO-treated cells bMECs (Figure 2B). To our knowledge, this is the first report indicating that octanoate modulates the TLR2 MA in NPPCs. A MCFA (lauric acid, 12C) induces the dimerization of TLRs (2 and 4) and activates the inflammatory response in macrophages (Lee et al., 2010). In agreement with this, our results support the hypothesis that 1 mM NaO activates bMECs via TLR2 before *S. aureus* challenge.

There is evidence suggesting that TLR2 is not a phagocytic receptor *per se*; nonetheless for bacterial recognition, it cooperates with other phagocytic receptors (i.e., scavenger receptors) (Mae et al., 2007). In a previous work, we reported that CD36 is involved in *S. aureus* internalization into bMECs but it has not a role in butyrate-reduced *S. aureus* internalization (Alva-Murillo et al., 2015). In contrast, we detected that CD36 is implied in 0.25 mM NaO-induced bacterial internalization (Figure 1C), but the CD36 MA or mRNA levels were not modified. In accordance, NaO (0.4 mM/24 h) does not modify the levels of CD36 mRNA in rat cardiomyocytes (Lockridge et al., 2008). However, NaO induces CD36 gene expression in bMECs, but this response was detected with a treatment of 10 mM NaO for 7 days (Yonezawa et al., 2004). In adipocytes, the expression of the CD36 gene is increased or down-regulated by this MCFA at 5 mM (8 days) or 1 mM (24 h), respectively (Guo et al., 2006; Suzuki et al., 2013). This led us to suggest that the modulation of CD36 depends on the concentration as well as exposure time of the treatment and is cell-type specific. Altogether, these results indicate that TLR2 and CD36 are involved in 0.25 mM NaO-induced *S. aureus* internalization, but only TLR2 is implied in the 1 mM NaO-reduced bacterial internalization.

Once TLR2 is engaged, some signaling pathways are triggered, where MAPKs and transcription factors are involved (Akira and Takeda, 2004). p38, JNK1/2, and ERK1/2 are implied in the *S. aureus* internalization into bMECs, and live bacteria induce JNK phosphorylation (Alva-Murillo et al., 2015). We observed that the phosphorylation levels of the MAPKs were down-regulated in 0.25 mM NaO-treated cells, and the activation of TFs was almost abolished (Figures 3–5). According to Kamata et al. (2007), the 1 mM NaO treatment up-regulated the MAPKs phosphorylation levels. In this sense, the 1 mM NaO-treated bMECs displayed a similar TFs profile than the unchallenged cells. However, the bMECs showed a slight activation of 13 TFs that are involved in IIR, but this response was reverted after *S. aureus* challenge (Figures 4E,F, 5). In a previous report, we showed that butyrate induces the activation of 8 TFs (Alva-Murillo et al., 2015), and this effect is stronger than the one provoked by NaO. To our knowledge, this is the first report that correlates this response with octanoate in NPPCs.

There are only few reports related to NaO and TF activation. The NaO attenuates the gene expression and protein levels of key adipogenic TFs, including PPAR, SREBP-1, and C/EBPα (Han et al., 2002; Guo et al., 2006). The protein levels of PPARγ, a TF with anti-inflammatory properties, increase in 10 mM NaO-treated bMECs (Yonezawa et al., 2004). Nevertheless, both concentrations of NaO used in this study reduced the basal activation of PPARγ (Figures 4C–E, 5B). In accordance, NaO (1–3 mM) in 3T3-L1 cells reduces the mRNA and protein level of this TF (Han et al., 2002; Guo et al., 2003). NaO inhibited the basal activation of NF-κB in bMECs (Figures 4–5). However, in Caco-2 cells, NaO does not modify the activation of NF-κB (Hoshimoto et al., 2002), but enhances the IL-1β-induced activation of NF-κB in intestinal epithelial cells (Andoh et al., 2000). On the other hand, NaO does not alter AP-1 activity in Caco-2 cells (Hoshimoto et al., 2002). Interestingly, some of the Ets-1 gene targets are *Fos* and *Jun*, which in turn forms AP-1 (Nagarajan et al., 2010), and we observed an increase of Ets-1 activation in the 1 mM NaO-treated cells, which could explain the induction of AP-1 observed in this condition (Figures 4E, 5A).

A previous report from our group showed the immunomodulatory ability of NaO (Alva-Murillo et al., 2013). In this sense, the antimicrobial response of bMECs treated with 1 mM NaO (24 h) is more effective than in 0.25 mM NaO-treated cells. We analyzed the gene expression of cytokines and chemokines in bMECs that might be modulated by *S. aureus* challenge (Lahouassa et al., 2007; Kim et al., 2011; Schukken et al., 2011; Alva-Murillo et al., 2014b). IL-1β is a pro-inflammatory cytokine that plays a critical role in the host defense against infection (Bannerman, 2009; Günther et al., 2011), and together with TNF-α is responsible for early inflammatory responses (Günther et al., 2011). We did not detect a significant change in IL-1β gene expression in the bMECs challenged with live *S. aureus* (MOI 30:1, 2 h) (Figure 6A), but the secretion of IL-1β in the *S. aureus*-challenged bMECs decreased (Figure 6B). Opposite to our results, Kim et al. (2011) showed that the level of IL-1β mRNA and the cytokine secretion were up-regulated in a bovine mammary epithelial cell line (MAC-T cell) stimulated with heat-killed *S. aureus* (10^8^ CFU/ml) for 3 h. The 0.25 mM NaO treatment slightly decreased the IL-1β secretion, and the infection alone did not change this effect. A similar result was observed at the mRNA level (Figure 6). On the other hand, the levels of IL-1β mRNA and cytokine secretion were up-regulated in the 1 mM NaO-treated cells. Interestingly, upon infection, these values were down-regulated.

IL-6 is a pleiotropic cytokine that acts as both a pro-inflammatory and an anti-inflammatory cytokine (Bannerman, 2009). This cytokine dominates the response toward *S. aureus* in bMECs (Günther et al., 2011). In this sense, *S. aureus* stimulation (MOI 10:1, 2 h) increases the IL-6 concentration in the MAC-T cell supernatant (Zheng et al., 2016). In agreement with this finding, we observed that the IL-6 secretion was augmented in *S. aureus*-challenged bMECs (Figure 6B), but the cytokine mRNA level was not altered. On the other hand, the IL-6 concentration in the supernatant of the 0.25 mM NaO-treated cells was similar to the basal levels, and this response was increased in the presence of *S. aureus* (Figure 6B). The 1 mM NaO treatment did not modify the IL-6 gene expression or the cytokine secretion. However, upon infection, the protein concentration in the supernatant was augmented even if the mRNA level was not altered. To our knowledge, there was no evidence that correlates NaO with IL-1β or IL-6 expression, but this is the first report indicating the modulation of both cytokines by this fatty acid.

IL-10 plays a central role in limiting inflammation, and exerts a broad anti-inflammatory effect on professional phagocytic cells by inhibiting the production of pro-inflammatory cytokines, chemokines, and eicosanoids (Bannerman, 2009). Our study demonstrated that the 0.25 mM NaO treatment induced IL-10 mRNA levels but it was down-regulated after infection (Figure 7A). In the 1 mM NaO-treated bMECs that were challenged with *S. aureus*, we observed an increase in IL-10 mRNA levels (~4-fold), showing an anti-inflammatory profile. To our knowledge, this is the first report indicating the regulation of IL-10 gene expression by NaO.

Chemokines, such as IL-8 and RANTES, recruit neutrophils from the blood to sites of infection, which is reflected by an increase of somatic cell count (SCC) in milk (Bannerman, 2009; Zbinden et al., 2014). The gene expression of both chemokines was not modified in the *S. aureus*-challenged bMECs (Figure 7B). In contrast, the expression of the IL-8 and RANTES genes are up-regulated in bMECs challenged with 10^7^ particles/ml of heat-killed *S. aureus* (3 h) (Günther et al., 2011). In the 1 mM NaO-treated bMECs, the IL-8 and RANTES mRNA levels were up-regulated, and after the challenge with *S. aureus*, the level of RANTES was slightly decreased, but the IL-8 gene expression was strengthened (Figure 7B). Evidence suggests that NaO modulates the inflammatory response at the intestinal level by showing a dichotomy. In intestinal epithelial cells treated with NaO (1–10 mM, 12 h), the IL-8 gene expression is not altered (Andoh et al., 2000). However, NaO strengthen the IL-1β-induced IL-8 gene expression and secretion (Andoh et al., 2000). On the other hand, in Caco-2 cells treated with NaO (1.3 mM, 24 h), the IL-1β-induced IL-8 secretion is suppressed (Hoshimoto et al., 2002). This demonstrates that NaO exerts either pro-inflammatory or anti-inflammatory activities. The discrepancies that we observed in the response modulated by NaO could be due to (i) the different concentration used, (ii) the time of incubation, (iii) the cell type used, and (iv) additional stimulus, such as *S. aureus* (live or killed). To our knowledge, this is the first study relating RANTES gene expression and its modulation by NaO.

We analyzed the promoter region of the cytokine genes evaluated in this study, as well as the AP genes analyzed previously to find putative transcription factor binding sites as a first step to establish a possible relation between the TF activation and the gene expression changes (Figure 8). Interestingly, all of the evaluated genes showed putative transcription binding sites for AP-1 and c-Myb, which might explain the AP and cytokine mRNA modulation by NaO, although, other mechanisms of regulation could exist in this model. It is known that one of the gene targets of Oct-1 is IL-8 (Sytina and Pankratova, 2003). We observed that the promoter region of this chemokine showed a putative Oct-1 binding site, and the activation of this TF in the 1 mM NaO-treated cells could be related to the up-regulation of the IL-8 gene expression (Figures 4, 5, 7B, 8). In addition, all the AP promoter regions showed a putative binding site for Ets-1. In accordance, Ets-1 modulates defensin expression in keratinocytes (Nagarajan et al., 2010). Although we noticed that all of the evaluated genes exhibited putative transcription binding sites for the NaO-activated TF, more experiments (i.e., luciferase reporter assays) are need to correlate the activation of TF and the gene expression.

Overall, our data support a model where NaO exerts an opposite and concentration-dependent response in bMECs. We observed that this effect is in relation to i) α5β1 integrin and TLR2 MA, (ii) MAPK (p38, JNK, and ERK1/2) phosphorylation, (iii) TF activation, (iv) the antimicrobial, and (v) the inflammatory response evaluated. The way in which NaO exerts this differential response on the modulation of the innate immune elements remains unknown. However, different factors could be involved, particularly the distribution of the fatty acid (extracellular or intracellular) that leads to different mechanisms of actions. In an extracellular manner, octanoate may activate cells through GPR109A and GPR84 membrane receptors, while intracellularly can bind to the TF PPARγ. Also, it is known that fatty acids (e.g., butyrate) regulate gene expression by epigenetic mechanisms (Tedelind et al., 2007). A recent report showed that NaO is a bone fide source of carbon (Acetil-CoA) for histone acetylation and exerts a concentration-dependent increase in histone acetylation in hepatocytes cells (1–5 mM, 24 h) modifying the epigenome, which is related to chromatin relaxation and gene expression (McDonnell et al., 2016). However, additional experiments are required to elucidate the contribution of these factors in the effects reported in this work.

## Conclusion

Altogether these results showed that NaO induces a concentration-dependent differential response on the innate immunity of bMECs. In this way, 0.25 mM abolishes the IIR by decreasing MAPK phosphorylation, TF activation, and the antimicrobial and inflammatory response, which favors *S. aureus* internalization into bMECs. On the other hand, bMECs are activated by 1 mM NaO via TLR2/p38/JNK/ERK1/2, and this improves the IIR and diminishes *S. aureus* internalization. Additionally, NaO (1 mM) might act as anti-inflammatory molecule during infection.

## Author contributions

NA, AO, and JL designed the experiments; NA performed the experiments; NA, AO, and JL analyzed the data; NA, AO, and JL wrote the paper.

## Funding

NA was supported by a scholarship from CONACyT. This work was supported by grants from CONACyT CB-2013-01 (221363) and CIC-UMSNH (14.5) to JL.

### Conflict of interest statement

The authors declare that the research was conducted in the absence of any commercial or financial relationships that could be construed as a potential conflict of interest.
